# Metal-Organic Frameworks (MOFs)-Based Nanomaterials for Drug Delivery

**DOI:** 10.3390/ma14133652

**Published:** 2021-06-30

**Authors:** Mohammad Reza Saeb, Navid Rabiee, Masoud Mozafari, Ebrahim Mostafavi

**Affiliations:** 1 Université de Lorraine, CentraleSupélec, LMOPS, F-57000 Metz, France; mrsaeb2008@gmail.com; 2 Department of Chemistry, Sharif University of Technology, Tehran 11155-3516, Iran; 3 Department of Tissue Engineering & Regenerative Medicine, Faculty of Advanced Technologies in Medicine, Iran University of Medical Sciences, Tehran 14665-354, Iran; m.mozafari@utoronto.ca; 4 Stanford Cardiovascular Institute, Stanford University, Stanford, CA 94305, USA; 5 Department of Medicine, Stanford University School of Medicine, Stanford, CA 94305, USA

**Keywords:** metal-organic frameworks, drug delivery, nanomedicine, biomedicine

## Abstract

The composition and topology of metal-organic frameworks (MOFs) are exceptionally tailorable; moreover, they are extremely porous and represent an excellent Brunauer–Emmett–Teller (BET) surface area (≈3000–6000 m^2^·g^−1^). Nanoscale MOFs (NMOFs), as cargo nanocarriers, have increasingly attracted the attention of scientists and biotechnologists during the past decade, in parallel with the evolution in the use of porous nanomaterials in biomedicine. Compared to other nanoparticle-based delivery systems, such as porous nanosilica, nanomicelles, and dendrimer-encapsulated nanoparticles, NMOFs are more flexible, have a higher biodegradability potential, and can be more easily functionalized to meet the required level of host–guest interactions, while preserving a larger and fully adjustable pore window in most cases. Due to these unique properties, NMOFs have the potential to carry anticancer cargos. In contrast to almost all porous materials, MOFs can be synthesized in diverse morphologies, including spherical, ellipsoidal, cubic, hexagonal, and octahedral, which facilitates the acceptance of various drugs and genes.

## 1. Strategies for Encapsulation of Cargo into MOFs

Metal-organic frameworks (MOFs), which are known to be extremely porous crystalline hybrid compounds (possessing a Brunauer–Emmett–Teller (BET) surface area in the range of ≈3000−6000 m^2^·g^−1^ [1]), are the result of a chemical alliance between an organic unit (i.e., mono-, di-, tri-, or tetravalent ligands, also known as struts or linkers) and an inorganic cluster/ion (i.e., transition or lanthanide metals). One of the advantages of MOFs is their versatility, meaning that their composition and topology are exceptionally tunable; moreover, they are extremely porous and represent an excellent BET surface area. A variety of methods, such as hydrothermal or solvothermal, mechanochemical, spray-drying, ultrasonic/microwave, electrochemical, and diffusion synthesis, have been employed in the development of a wide variety of MOFs that possess the potential for myriad purposes, including energy storage, catalysis, sensor, nonlinear optics, protection of metals against corrosion, semiconductors, solar conversion of fuels, and biomedical applications. In particular, during the past decade, nanoscale MOFs (NMOFs), as cargo nanocarriers, have played an essential role in medicine and biomedical engineering. Compared to other nanoparticle-based delivery systems (such as porous nanosilica, nanomicelles, and dendrimer-encapsulated nanoparticles), NMOFs are more flexible, provide better biodegradability, can be more easily functionalized to meet the required level of host–guest interactions, and offer a wider range of pore size. As a result, they are better candidates to carry anticancer cargos. In contrast to almost all porous materials, such as nanosilica [2,3], MOFs can be synthesized in wider range of diverse morphologies with a controlled microstructure, including spherical, ellipsoidal, cubic, hexagonal, and octahedral, which facilitates the acceptance of various drugs and genes. Moreover, fluorescence agents and organic dyes can be encased in MOFs for imaging, photothermal therapy (PTT), and photodynamic therapy (PDT) [4].

**Scheme 1:**
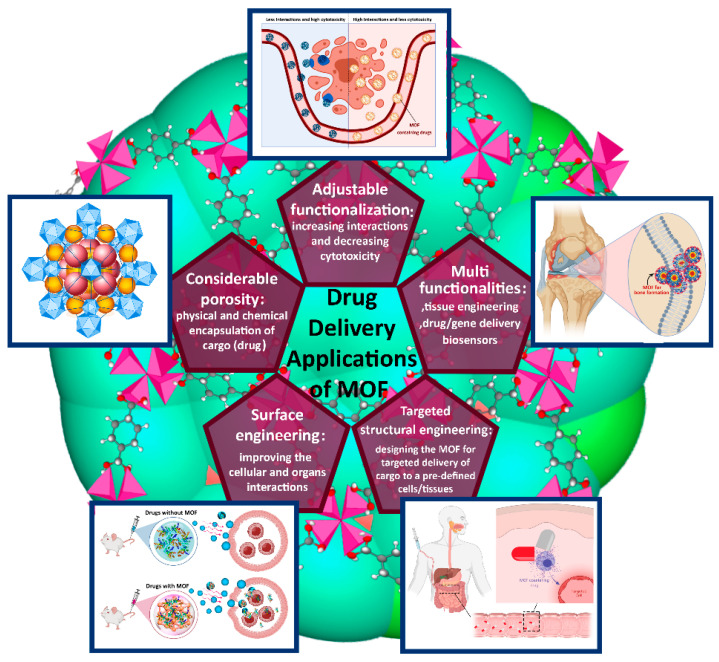
A schematic illustration of the main applications of MOFs in drug delivery.

The present perspective discusses different properties of MOFs, such as their physical, chemical, and biomedical aspects, in drug delivery systems (DDS). Scheme 1 provides a short overview of the applications of MOFs in drug delivery. Stock and Biswas comprehensively reviewed the effect of the synthesis route on the topology and morphology of MOFs [5]. For instance, microwave-assisted methods mainly result in nanoscale particles of MOFs. Nevertheless, the correct choice of metal determines the efficiency of drug delivery. In addition, for efficient drug delivery, it is essential to functionalize NMOFs (e.g., with –NH_2_). The presence of unreacted linkers in the framework is inevitable in as-synthesized NMOFs. Under such circumstances, the pore dimension significantly depends on the flexibility of the framework. For example, in the case of X- Matériaux de l’Institut Lavoisier (MIL)-53 (X = Fe, Al, Cr), there is a large pore size window by exchange of metals, such that variance in the unit cell volume of up to 60% is observed with no framework topology change. The most widely used MOFs for drug delivery are Fe metal-based MOFs with bio-organic linkers (MILs; 53, 88, 100, and 101) having a pore size in the range of ca. 7–12.5 nm [6]. A large number of pores, channels, and cavities in MOFs provide the therapeutic agents with excellent carriers for the delivery of proteins, drugs, genes, Deoxyribonucleic acid (DNA), and ribonucleic acid (RNA) through covalent and non-covalent interactions [7,8]. Nevertheless, optimizing pore size for controlled release behavior depends on several parameters, mainly the interaction between drug and framework.

Three kinds of cargo loadings or encapsulation strategies have systematically been applied for the encapsulation of therapeutic agents (such as chemically-synthesized small molecules, botanically available molecules, biotherapeutic macromolecules, and nucleic acids-based therapeutic agents) in MOFs; these are classical (direct assembly), modern (post-synthesis), and mixed strategies. The encapsulation strategy can be selected based on the state of the cargo–MOFs interactions. The direct assembly strategy has a one-pot nature in which the cargo participates in the MOF formation in situ through its coordinated functions that bond to the metal ligands. Facile synthesis of nanoparticles having various size and morphology (e.g., nanoparticles with approximately 40 nm, nanorods of 80 nm × 80 nm× 1000 nm, and nanoplates around 100–200 nm), proper distribution, and high loading of cargos (such as pamidronate (Pam), zoledronate (Zol), doxorubicin hydrochloride (DOX), *c*,*c*,*t*-(diamminedichlorodisuccinato) Pt (IV) (DSCP), PTT, PDT) to the NMOFs (≈60–75 wt.%) are the main advantageous features of this therapeutic strategy. However, the delivery performance of this strategy is governed by the kinetics of cargo decomposition in the biological environment. MOFs, including Materials of Institute Lavoisier (MIL)-100, Zeolite imidazolate framework (ZIF)-8, and University of Oslo (UiO)-66, are the most popular structures employed for direct assembly encapsulation. Unlike the direct assembly approach, the post-synthesis strategy is a two-step synthesis. First, the desired NMOF structure having a defined size, morphology, and characteristics is synthesized. Second, the cargo is encapsulated in NMOFs with the aid of coordinative metal sites, functional sites of the struts, or the defect in the ligands of the metal nodes. Recently, some molecules named “modulator” (e.g., monocarboxylic acid) have been used to facilitate the formation of the defect in the ligands that substantially enhances the ability of NMOFs to accept and release cargos. Although the resulting spherical NMOFs (having a diameter of ca. 70–180 nm) can host some cargos (diiodo-substituted BODIPYs (I2-BDP), oligohistidine-Tags (His-Tags) peptides or proteins, phosphate-modified DNA) in a more controlled manner compared to NMOFs prepared via direct assembly, the loading level of the latter strategy is relatively low (3–25 wt.%) [9,10,11]. MOFs, including MIL and UiO, are most prevalently developed by post-synthesis encapsulation. It is now well understood that metastatic tumor and cancer progression unavoidably occur by the alteration of the metabolism, in addition to uncontrolled mutation and hypoxia-induced angiogenesis [12,13]. This situation demands an efficient delivery system that represents synergistic cargo encapsulation and release, e.g., the cargo should overcome the blood–brain barrier (BBB). The mixed strategy makes good use of both classical and modern approaches, such that nanorods (50–100 nm length and 20–30 nm width) with an intermediate loading capacity (40–60 wt.%) are formed. The NMOF structure developed by this method can encapsulate inhibitors, indoleamine 2,3-dioxygenase (IDOi), (5,10,15,20-tetra(p-benzoato) chlorine (H_4_TBC), olsalazine, and phenethylamine. MOFs, including TBC-Hf and Mg_2_(olz), are the most popular structures used in mixed encapsulation. Examples also exist of multivariate modulation strategies, which permit the loading of two or three kinds of drugs in MOFs, e.g., Zr MOF UiO-66 [14].

## 2. Stimuli-Responsive MOFs for Cargo Delivery

Stimuli-responsible MOFs are a class of nanomaterials that can be treated in single-stimuli-responsive and multi-stimuli-responsive nanocarriers. They can not only encapsulate cargos at the desired level depending on the synthesis and encapsulation strategy, but also respond monotonically to the stimuli, such as temperature, pH, ion, magnetic field, and pressure. The pH-responsive frameworks are the most common among single-stimuli-responsive MOFs for cancer therapy, bearing in mind the sensitivity of MOFs to the pH and the acidity of the tumor microenvironment. Some examples of pH-responsive structures are poly(acrylic acid)@zeolitic imidazolate framework-8 (PAA@ZIF-8), DOX incorporated silica-supported 1,1′-(1,4-butanediyl) bis (imidazole) (bbi), and DOX/Fe(bbi)@SiO_2_–FA, UiO-66, and, more recently, gadolinium (III)-based MOFs. In some cases, the regulation of pH may allow for 100% release of cargos, an advanced feature of pH-sensitive MOFs. ZIF-8, UiO-66, porous coordination network (PCN)-221, and Zhejiang University (ZJU)-101 are examples of pH-responsive MOFs. Magnetically responsive MOFs are designed to deliver cargos under magnetic fields, e.g., magnetic hyperthermia and magnetic resonance imaging (MRI) [15,16,17]. A feature of this therapeutic approach is the ability to apply multi-stage delivery of drugs over a predesigned release period. Fe_3_O_4_-Cu_3_(BTC)_2_, and ϒ-Fe_2_O_3_-MIL-53 (Al) are examples of MOFs with a magnetic-responsive characteristic. Ion-responsive MOFs are engineered nanostructures grounded on a physicochemical concept. In such structures, the penetration of the cargo from MOFs to the biological environment is fueled by the electrostatic interactions between the MOF and the cargo. BioMOF-1 and MOF-74-Fe (III) are examples of MOFs possessing an ion-responsive characteristic. Temperature-responsive MOFs are sensitive to heat and appear to be promising nanocarriers for cancer therapy. The release of cargo takes place at a specified temperature, and targeted delivery from MOFs is attained by temperature regulation. UiO-66-Poly(N-isopropylacrylamide) (PNIPAM), ZJU-63-CH_3_, and generally temperature-sensitive polymer-coated MOFs are examples of temperature-responsive MOFs. Engineered MOFs should avoid accelerated delivery of the drug before being exposed to the target tissue [18,19]. This can be achieved by pressure-responsive MOFs; for instance, ZJU-800 is studied as a pressure-sensitive MOF. The multiplicity of parameters contributing to the success or failure of a DDS is the main reason behind developing innovative multiple-stimuli-responsive MOF structures. Fe-BTC@Zn-BTC, CP5-capped UMCM-1-NH-Py, CP5-capped UiO-66-NH_2_, PEG-RGD-βCD-SS-MIL-101, and βCD-capped UiO-68-azo are examples of multiple-stimuli-responsive MOFs. Such systems are still immature and should be optimized for a targeted delivery mission [20,21].

## 3. Functionalization of MOFs for Cargo Delivery

Because MOFs are highly porous and have a very high surface/volume ratio with extremely ordered structures, their cavities and lateral surfaces can more potently react with functional groups [22,23]. This can be carried out in situ or through functionalization of the pre-MOF structure. The functionalization of MOFs provides the therapeutic agents with a higher loading capacity. Typically, surface adsorption, pore encapsulation, covalent binding, and the use of the functional molecules as the building blocks are the possible approaches for the functionalization of MOFs. Functional molecules can be easily adsorbed on the MOF surface, given their highly porous structure [24,25]. This phenomenon is supported by the hydrogen bonding, van der Waals interactions, and π–π interactions, as applied in enzyme immobilization. UiO-66-NH_2_, ZIF-8, and Ni-IRMOF-74-II MOFs are developed for the surface adsorption process. Pore encapsulation is also used to functionalize MOF, as an in situ process in which larger molecules (functional molecules) are trapped in a highly porous structure of the MOF, and functionalization occurs based on immobilization. MP-11@Tb-mesoMOF and different bioconjugate forms of PCN-333 MOFs are obtained through pore encapsulation [26,27,28]. Covalent bonding is the third method implemented in MOF functionalization, and MIL-88B (Cr), MIL-101 (Cr), and Zr-based MOFs are a few examples of functionalized MOFs synthesized by this method. Organic linkers and inorganic metal clusters can form covalent bonds in the MOF structure. The click reaction is an example of this method. The functional molecules are the building blocks of MOFs for the synthesis of bio-based MOFs, in which amino acids, peptides nucleobases, and saccharides are examined as organic ligands. Zn_8_(ad)_4_(BPDC)_6_O·2Me_2_NH_2_·8DMF·11H_2_O (bioMOF-1), and (Zn_8_(ad)_4_(BPDC)_6_O_2_·4Me_2_NH_2_·49DMF·31H_2_O) (bioMOF-100) MOFs are obtained through this process [4,29].

## 4. Applications of MOFs in Drug Delivery

Thus far, several nanomaterials have been applied in DDS, mainly carbon-based nanostructures including reduced graphene oxide (rGO), multi-walled carbon nanotube (MWCNT), and different types of natural and synthetic polymers. Despite their relative cell viability and low immune response inside the microorganisms, they have poor stability, lack the ability of modifications, and have confined porous structures. Inorganic-based nanomaterials, especially MOFs, emerged as a new material for DDS with improved properties compared to the conventional DDS. MOFs are not only promising due to their physicochemical structures and morphology, but also due to their ability to provide a wide range of interactions on the surface or inside the porosities and interconnected channels. For instance, MOFs can provide full physical interaction with the cargo by synthesizing free-functional groups such as UiO-66; or considerable π–π interactions between the host (MOFs) and the guest (cargos) in the synthesis of π-rich MOFs.

Moreover, MOFs are highly stable and well-organized 3D nanostructures with the potential for facile pore engineering by manipulating synthesis routes. Increasing the temperature by 10–50% results in an increase in the pore size and pore volume at the rate of 7–62%. Further, other parameters, including pH, the speed of the stirrer, solvent(s), and the purity of precursors, could change the pore size, pore volume, surface area, and even the size and zeta potential. Therefore, normal and even stimuli-responsive MOFs could be used in targeted DDS, suitable for different cell lines, organs, and targeted tissues by changing synthesis parameters. From another perspective, modifying the surface and inside of the porosities of MOFs could provide a wide range of interactions with the cargos, ranging from drugs to sensitizers and genetic materials (Table 1). By changing the surface functionality from hydroxides to amine(s), amide(s), and imide(s), the payload efficiency of the selected drug increases by an average rate of 38%. Moreover, natural polymers and leaf extracts on the surface of Cr, Zr, and Zn MOFs can enhance the relative cell viability by the rate of 2–14%, improve stability up to 150 h, and provide considerable pH tolerance (3.5 < pH < 9) [30,31,32].

## 5. Conclusions, Challenging Features, and Future Perspectives

MOFs are progressively developing from generation to generation to meet the requirements for biomedical applications. MOFs are excellent nanocarriers, but their size, shape, functionality, and loading capacity should be further controlled or optimized for a particular application. In recent years, the green synthesis of MOFs for biomedical applications has been the center of attention. This is because green MOFs are inherently sustainable with acceptable biocompatibility. Nevertheless, they have a limited level of stability in aqueous media and deteriorate at high temperatures. Therefore, their potential as cargo for encapsulation and DDS should be further enhanced. Currently, there is global concern regarding the situation caused by the SARS-CoV-2 virus or COVID-19 pandemic. Several companies commercialized their products to vaccinate people against SARS-CoV-2/COVID-19 [50]. Nevertheless, millions of people globally are struggling to survive, mainly because of the inability to access vaccines, the regional mutations of COVID-19, and/or the ineffectiveness of the vaccine. Thus, vaccines still need to be optimized to enhance their protection window and lessen their side-effects for long-term immunity to COVID-19 infection. Even in the range of ppm, some side effects, such as thrombosis, raise serious concerns about COVID-19 vaccines. The rapid production of mRNA vaccines during the COVID-19 pandemic encoded for the protein of SARS-CoV-2 can be responded to via the design of multi-responsive nanoparticles. Bio-MOFs are a promising class of MOFs, which can be prepared using biological endogenous organic ligands, e.g., amino acids, peptides, proteins, porphyrins, and saccharides. It is speculated that mRNA can be more stable and resistant to RNase-mediated degradation if it is complexed with positively charged bio-MOFs, which can further form self-assembled virus-sized particles suitable for administration through different routes. During the endocytose process, the Bio-MOFs can facilitate endosomal escape and deliver the genetic cargo in the cytosol, where the mRNA is translated into antigenic proteins, forcing the immune system machinery to produce the targeted antibodies. A current drawback of nanoparticulate delivery of vaccine formulations is their long-term storage limitations, which create logistical challenges to their prospective distribution and administration. Bio-MOFs might be of great interest to those working on the concept of vaccine delivery through nanomedicine strategies due to their biocompatibility, nano-size structure, and physicochemical characteristics, which can protect the vaccine cargos from degradation, and suggest controlled biodistribution and intracellular localization and release of the vaccine. We believe that MOFs can contribute to enlightening the future perspective for developing highly sophisticated and critical tasks in the field of drug delivery. The current state of the literature indicates that green MOFs have neither been comprehensively reviewed nor systematically classified. Moreover, the selection of green multivalent ligands and struts together with transition metals remain challenging aspects of MOF synthesis for drug delivery applications.

## Figures and Tables

**Table 1:** A literature survey of the MOF-based nanomaterials for cargo delivery.

MOF-Based Nanomaterial	Cargo	Function	Essential Components	Targeted Cell Line(s)	Disadvantages	Refs.
MIL-101-NH_2_@silica shell@RGDfk	Cisplatin	Chemotherapy, Optical imaging, Targeted drug delivery,	1,4-BDC-NH2, Fe^3+^, Br-BODIPY, silica shell	HT-29	toxicity in higher concentrations, Not green, complex synthesis method	[33]
Zn-H_2_BDP	Mitoxantrone	Sustained drug release in more sophisticated conditions, Chemotherapy, and reducing toxicity	Zn^2+^, H_2_BDP	J774	Not applicable for in vivo experiments, not green	[34]
Mn-bisphosphonate@peg-AA	Zoledronate	Magnetic resonance imaging (MRI) contrast agent, chemotherapy	DOPA, DSPE, DOPC, anisamide, Mn^2+^	AsPC-1, MCF-7	Expensive, Not green, toxic in some higher concentrations	[35]
Zn-TATAT	5-Fu	Sustained drug release in more sophisticated conditions, Chemotherapy, and reducing toxicity	TaTAT, Zn^2+^	-	Considerable aggregations in most of the concentrations, limited usage for in vivo experiments	[36]
UiO-67@ and UiO-66@PCL	Taxol, Cisplatin	Sustained release of drug in a limited condition, Chemotherapy, and reduced toxicity	UiO-67, UiO-66, Polycaprolactone, Polyethylene glycol	HSC-3, U-87MG	high-temperature of synthesis, not green, drastic toxicity in some concentrations, not cost-effective	[37]
UiO-66@Fe_3_O_4_	DOX	Reduced toxicity, Chemotherapy	1,4-BDC, ZrCl_4_, Fe_3_O_4_	3T3, HeLa	Not green, limited utilization, non-sustained release	[38]
MIL-100-Hep	Caffeine	Chemotherapy, limited inflammation response	1,3,5-BTC, Fe^3+^, Rhodamine, Heparin	HL60, J774.A1	Not green, not sequential drug release, limited usage, difficult synthesis method	[39]
ZIF-90@Fe_3_O_4_	5-Fu	Chemotherapy, MRI, Magnetic thermal delivery	Rat serum albumin, Zn^2+^, Fe_3_O_4_, Polyvinylpyrrolidone (PVP)	-	Not green, considerable cytotoxicity, limited application	[40]
ZIF-8-Au_25_@Fe_3_O_4_	-	Targeted delivery, MRI, chemotherapy, photodynamic therapy	Fe_3_O_4_, Zn^2+^, HMelM	HeLa L929	Not green, considerable toxicity, harsh synthesis condition, limited sustained release	[41]
IRMOF-3@FA-RITC@Fe_3_O_4_	Paclitaxel	Optical imaging, MRI, Chemotherapy	Folic acid, PVP, Fe_3_O_4_, Zn^2+^, rhodamine B isothiocyanate	NIH3T3, HeLa	Not green, difficult synthesis method, very low yield of synthesis, toxic	[42]
MIL-100@C@Fe_3_O_4_	Dihydroxyacetone (DHA), Fe(III)	MRI, pH-responsive delivery, optical imaging	Fe_3_O_4_, Fe^3+^, ferrocene, 1,3,5-BTC	HeLa, A549	Not green, low yield of synthesis, harsh synthesis method	[43]
Mn coordination polymers	Non-steroidal anti-inflammatory drugs (NSAIDs)	Drug delivery, cell imaging	NSAIDs, Mn(ClO_4_)_2_	RAW264.7	Not green, complicated synthesis procedure, not scalable, limited toxicity	[44]
UiO-PDT	-	Photodynamic therapy	12-BDP, 1,4-BDC, ZrCl_4_	CT26, C26, B16F10	Not green, limited toxicity, expensive synthesis method	[45]
TCPP-Hf-PEG	-	Radiation therapy, photodynamic therapy	Meso-Tetra(4-carboxyphenyl)porphine (TCPP), HfCl_4_, polyethyleneglycol (PEG)	NIH3T3, HeLa, 4T1	Not green, limited cell viability, limited in vivo applications	[46]
MIL-10@PB	Artemisinin	Optical imaging, MRI, Chemotherapy	PVP, 1,3,5-BTC, Fe^3+^	HeLa	Not green, complex synthesis procedure, relatively low cell viability	[47]
p(HEMA)-GMA-UiO-66	DOX and pCRISPR	Targeted drug/gene delivery, sustained-release in tissue and cells, stimuli-responsive delivery, lowest cytotoxicity, fully biocompatible	UiO-66, Glycidyl methacrylate (GMA), (Hydroxyethyl)methacrylate (HEMA)	HepG2, HeLa, HEK-293, PC12	Limited in vivo applications	[48]
Fc-Gd@SiO_2_(RBITC)-RGD	-	Targeting, MRI	Fc, Gd^3+^, RBITC, silica	MCF-7, U87MG	Not green, limited in vivo usage, considerable cytotoxicity	[49]
p(NIPAM)-GMA-UiO-66	DOX and pCRISPR	Targeted drug/gene delivery, sustained release in tissue and cells, stimuli-responsive delivery, lowest cytotoxicity, fully biocompatible	UiO-66, GMA, NIPAM	HEK-293, HeLa, HepG2, PC12	Limited in vivo applications	[48]
